# Phage Display-Derived Monoclonal Antibodies Against Internalins A and B Allow Specific Detection of *Listeria monocytogenes*

**DOI:** 10.3389/fpubh.2022.712657

**Published:** 2022-03-15

**Authors:** Gustavo Marçal Schmidt Garcia Moreira, Sabine Gronow, Stefan Dübel, Marcelo Mendonça, Ângela Nunes Moreira, Fabricio Rochedo Conceição, Michael Hust

**Affiliations:** ^1^Technische Universität Braunschweig, Institut für Biochemie, Biotechnologie und Bioinformatik, Abteilung Biotechnologie, Braunschweig, Germany; ^2^Leibniz Institute DSMZ-German Collection of Microorganisms and Cell Cultures, Braunschweig, Germany; ^3^Universidade Federal do Agreste de Pernambuco, Curso de Medicina Veterinária, Garanhuns, Brazil; ^4^Laboratório de Imunologia Aplicada, Núcleo de Biotecnologia, Centro de Desenvolvimento Tecnológico, Universidade Federal de Pelotas, Pelotas, Brazil

**Keywords:** *Listeria monocytogenes*, monoclonal antibody, internalin A, internalin B, phage display, detection, food safety

## Abstract

*Listeria monocytogenes* is the causative agent of listeriosis, a highly lethal disease initiated after the ingestion of *Listeria*-contaminated food. This species comprises different serovars, from which 4b, 1/2a, and 1/2b cause most of the infections. Among the different proteins involved in pathogenesis, the internalins A (InlA) and B (InlB) are the best characterized, since they play a major role in the enterocyte entry of *Listeria* cells during early infection. Due to their covalent attachment to the cell wall and location on the bacterial surface, along with their exclusive presence in the pathogenic *L. monocytogenes*, these proteins are also used as detection targets for this species. Even though huge advancements were achieved in the enrichment steps for subsequent *Listeria* detection, few studies have focused on the improvement of the antibodies for immunodetection. In the present study, recombinant InlA and InlB produced in *Escherichia coli* were used as targets to generate antibodies via phage display using the human naïve antibody libraries HAL9 and HAL10. A set of five recombinant antibodies (four against InlA, and one against InlB) were produced in scFv-Fc format and tested in indirect ELISA against a panel of 19 *Listeria* strains (17 species; including the three main serovars of *L. monocytogenes*) and 16 non-*Listeria* species. All five antibodies were able to recognize *L. monocytogenes* with 100% sensitivity (CI 29.24–100.0) and specificity (CI 88.78–100.0) in all three analyzed antibody concentrations. These findings show that phage display-derived antibodies can improve the biological tools to develop better immunodiagnostics for *L. monocytogenes*.

## Introduction

The *Listeria* genus contains a total of 20 species, from which 14 have been described in the last decade (1). Among these, *Listeria monocytogenes* is the major causative agent of listeriosis in humans. This species has 13 different serovars, from which the ones named 4b, 1/2a, and 1/2b are responsible for at least 95% of the infections (2). Due to the ubiquitous nature of this Gram-positive, facultative anaerobic, non-sporulating, bacterium it is virtually impossible to eliminate it from certain environments. This is a problem in particular for food production facilities since the resistance to a broad range of pH, salt concentration, biofilm formation, and temperature change allows this bacterium to be often present in food, especially those uncooked or ready-to-eat products (3–5).

The recent description of many *Listeria* species already indicates the growing importance of studies about this bacterial genus (1, 6–8). In addition, despite the food safety surveillance performed in Europe and the USA, there is still a considerable number of outbreaks being reported. In 2021 alone, one outbreak was reported in the USA, with 1 death amongst 11 infected people (9), while in Europe a surveillance report indicates that listeriosis will tend to increase in the coming years after 2,502 cases and 222 deaths were described across several European countries in 2017 (10).

As a foodborne disease, the development of listeriosis requires the bacterial cells to reach the gastrointestinal tract to start the infection. Receptors on intestinal cells are recognized by a group of proteins on the bacterial surface that promotes entry into the host's cells (11). The main bacterial proteins involved with this host's cell invasion are internalins A (InlA) and B (InlB), which are covalently attached to the cell wall and are only present in members of the pathogenic species *L. monocytogenes* and some strains of *Listeria ivanovii* (12). Therefore, as these proteins play a major role in pathogenesis and are easily accessed on the bacterial surface, they are the most studied targets on pathogenesis and development of biological reagents for the detection of *Listeria*.

The standard *Listeria* detection is based on microbiological culture and biochemical characterization, which involve an initial enrichment step followed by the detection itself (13). Although this method is very precise, it takes about seven days from sample collection to result, which often is too slow for monitoring many food production processes. To overcome this problem, strategies to improve the enrichment of bacteria from samples have been successful, such as the use of a Pathogen Enrichment Device (PED) (14). For the following detection step, molecular techniques such as PCR have also shown a good performance, although it requires trained personal, and specialized infrastructure to perform the tests, e.g., thermocycler, fluorescence detector, and electrophoresis apparatus (15). To overcome these limitations for *Listeria* detection, immunodetection with ELISA or lateral flow-based techniques, which rely on specific and sensitive monoclonal antibodies (mAbs), offer a way for improvement.

While the recent years brought significant advancements on the enrichment step for *Listeria* identification, the generation of suitable mAbs for *Listeria* detection has not been addressed accordingly. Most of the molecules already described relied on classical hybridoma technology for their production, which, although useful, is often limited in providing high number of potentially useful binders (16–18). In contrast, phage display explores a vast repertoire of binders using a library-based system for acquiring antibodies (19) and, thus, is a technique that allows the generation of antibodies against virtually any target when using a naïve antibody phage library, the so-called “single-pot” antibody libraries (20). However, this procedure has been scarcely explored for the generation of suitable antibodies for *Listeria* detection.

In addition to the detection of the pathogenic *Listeria* species, distinction from the non-pathogenic species is also important, as the same food sample source may contain both of them (21). This way, it has been described that non-pathogenic species, mainly *Listeria innocua*, can overgrow *L. monocytogenes*, leading to potentially false-negative results (21). To avoid these unwanted results, targets present in all *Listeria* species, such as flagellin (18) or FBA (22) can be used. Aligned with this, we have previously described the new biomarker pyruvate dehydrogenase complex–enzyme 2 (PDC-E2) for *Listeria* spp., and used phage display for the generation of antibodies against it, which allowed the detection of all tested *Listeria* species (23).

The aim of this study was to generate recombinant antibodies against the targets internalin A (InlA) and B (InlB), which are known to be involved in *Listeria* pathogenesis. These proteins are mainly present in *L. monocytogenes* and, thus, are capable of providing species-specificity. In line with this, four recombinant mAbs against InlA and one against InlB showed high sensitivity and specificity when tested against the three most prevalent *L. monocytogenes* serovars (4b, 1/2a, and 1/2b), as well as against most of the remaining *Listeria* spp. and 15 non-*Listeria* species. Thus, the combination of antibodies against InlA and InlB with those previously described against *Listeria* spp. could be employed to perform tests that distinguish pathogenic from non-pathogenic *Listeria* in parallel.

## Materials and Methods

### Expression in *Escherichia coli*, and Purification of Recombinant InlA and InlB

The plasmids pAE-*inlA* and pET28a-*inlB* for the recombinant protein expression of InlA and InlB in *E. coli*, respectively, were constructed and described previously (24, 25). *E. coli* BLR(DE3) (Novagen) containing the individual plasmids was grown in Luria-Bertani (LB) broth with 100 μg/mL ampicillin for 16 h at 37°C while shaking at 200 RPM. Cells were inoculated in 500 mL LB and grown until OD_600_ = 0.5–0.8 before IPTG was added to a final concentration of 0.15 mM. Expression was induced for 4 h under the same conditions before the cells were harvested (16,000 x g, 10 min, 4°C), suspended in lysis buffer (Tris 20 mM, NaCl 0.5 M, Imidazole 5 mM, pH 7.9), sonicated, and the supernatant was used for Ni^+2^-affinity purification using Ni-Sepharose (GE Healthcare). Purification washing was performed with the same buffer containing 20 mM Imidazole, while elution contained 500 mM imidazole. Eluted fractions were quantified via spectrophotometry at 280 nm, pooled, and dialyzed against phosphate-buffered saline (PBS) using a previously described protocol for the gradual removal of NaCl and Imidazole (26).

### Antibody Panning on Purified rInlA and rInlB and Monoclonal scFv Screening

For the generation of antibodies against rInlA and rInlB, one well of an ELISA Costar plate was coated with 1 μg of the protein diluted in 150 μL of PBS. In parallel, one additional well was coated with Panning Block solution [1% (w/v) skimmed milk powder, 1% (w/v) BSA, diluted in PBS containing 0.05% (v/v) Tween20] for pre-incubation. The remaining procedure was identical to that described elsewhere (27) with libraries HAL9 and HAL10 (28) mixed in the same panning well, using three panning rounds. The strain *E. coli* TG1 was used to perform the panning rounds, while *E. coli* XL1-Blue MRF' was used to produce soluble scFv in 96-well plates. Screening of soluble monoclonal scFv in ELISA was performed as previously described, using *L. monocytogenes* ATCC 7644, and *Bacillus subtilis* 168 NCIB 10106 as living bacteria coated onto the plates. The latter species was chosen because *Bacillus* is phylogenetically related to *Listeria* (29), and, thus, could provide a different set of antigens as negative control of the binders. Additional plates coated with 200 ng/well of either the respective recombinant protein or BSA, diluted in PBS, were used as a positive and negative control, respectively.

### Production of scFv-Fc in HEK293 Cells, Immunoblot and Indirect ELISA

The scFv binding specifically to *L. monocytogenes* in the screening ELISA were sequenced and subcloned into pCSE2.6 vector (30) for expression in Expi293F (Thermo Scientific). The scFv-Fc were produced with mouse IgG2a Fc using a previous protocol (31). When using this expression vector, the Fc region does not cause a change in the binding properties even though it does not have the same species of origin as the scFv (30). Thus, the murine Fc was chosen because the potential application of this mAbs would require mouse Fc detection and because there was no plan to use them for human treatment. Later, the mAbs were purified with protein A-affinity and buffer exchanged to PBS. The applicability of the antibodies in immunoblot was tested after running 1 μg of recombinant protein in a 12% SDS-PAGE, and transferring the proteins to a PVDF membrane (Roth) activated with methanol. The membrane was blocked with 2% (w/v) skimmed milk powder diluted in PBS with 0.05% (v/v) Tween20 (2% MPBS-T) for 16 h at 4°C. Afterwards, 1 μg/mL of each of the scFv-Fc was diluted in blocking buffer and incubated for 1 h at RT, followed by the incubation of goat anti-mouse IgG Fc-specific HRP-conjugated (1:40,000; Sigma), and DAB solution (6 mg 3.3-diaminobenzidine tetrahydrochloride; 10 μL 30% (v/v) H_2_O_2_; 9 mL PBS; 1 mL NiSO_4_ 250 mM) development. The indirect ELISA procedure was similar to the screening with additional use of *Listeria innocua* DSM 20649 as negative control strain (preparation of living cells suspension is detailed in the next topic). This time, the scFv-Fc were diluted in 2% MPBS-T using √10-fold series and incubated for 1 h at RT, followed by incubation with the same secondary antibody from the immunoblot.

### Indirect ELISA for *Listeria* spp. Detection

The bacteria included in this study were acquired from the Leibniz Institute DSMZ-German Collection of Microorganisms and Cell Cultures, which gives a catalog number (DSM) to every strain of its collection (Table 1). They were cultured in BHI, except for *Lactobacillus paracasei*, for which MRS medium was used, according to the recommendations of the strain manuals. The assay procedure, dilution of mAbs, and list of species were as described (23). Briefly, cells were cultured, washed with PBS, suspended in Carbonate-Bicarbonate 150 mM, pH 9.6, to OD_600_ = 1.0, and coated onto Costar ELISA plates (Corning). Antibodies were diluted in 2% MPBS-T in three concentrations: (1) the approximate EC_50_ (EC_50_); (2) a √10 dilution above the EC_50_ (EC${}_{50^{+}}$); and (3) a √10 dilution below the EC_50_ (EC_50_-). Goat anti-mouse IgG Fc-specific HRP-conjugated (1:30,000; Jackson ImmunoResearch Laboratories) was used as the secondary antibody. Each antibody concentration was tested twice against each bacterial strain in individual plates. However, only one of these plates was used for the statistical analysis. A signal-to-noise ratio was calculated for each well by using the signal of secondary antibody alone against the bacteria as a reference for noise. The data were analyzed with GraphPad software (Prism, v 5.01) for the Receiver Operating Characteristic (ROC) determination. This way, the sensitivity, specificity, and confidence intervals (CI) were obtained.

**Table 1:** List of the *Listeria* and non-*Listeria* species used in indirect ELISA for diagnostic performance assessment.

**Species**	**Serovar**	**DSM**
*L. monocytogenes*	4b	15675
	1/2a	102976
	1/2b	19094
*L. innocua*	6a	20649
*L. marthii*	NI	23813
*L. welshimeri*	1/2b	20650
*L. ivanovii* subsp. *ivanovii*	5	20750
*L. seeligeri*	1/2b	20751
*L. floridensis*	NI	26687
*L. fleischmannii subsp. fleischmannii*	NI	24998
*L. aquatica*	NI	26686
*L. grayi*	NI	20601
*L. cornellensis*	NI	26689
*L. rocourtiae*	NI	22097
*L. booriae*	NI	28860
*L. riparia*	NI	26685
*L. weihenstephanensis*	NI	24698
*L. grandensis*	NI	26688
*L. newyorkensis*	NI	28861
*Salmonella enterica* subsp. *enterica*	Typhimurium	17058
*Escherichia coli*	O157:H7	17076
*Pseudomonas aeruginosa*	NI	50071
*Klebsiella pneumoniae*	3	30104
*K. aerogenes*	NI	30053
*Enterobacter cloacae* subsp. *cloacae*	NI	30054
*Staphylococcus aureus*	3	20231
*Jonesia denitrificans*	NI	20603
*Bacillus subtilis*	NI	10
*B. thuringiensis*	NI	2046
*B. cereus*	NI	31
*Enterococcus faecium*	D, 11	20477
*E. faecalis*	D	20478
*E. lactis*	NI	23655
*Lactococcus lactis*	N	20481
*Lactobacillus paracasei* subsp. *paracasei*	NI	5622

*NI, not informed*.

*DSM, collection number from Leibniz Institute DSMZ-German Collection of Microorganisms and Cell Cultures*.

## Results and Discussion

### Panning With Human Naïve Antibody Library Over rInlA and rInlB Provides Binders Reactive in Immunoblot and ELISA

For antibody selection, the human naïve antibody gene libraries HAL9 and HAL10 were used. After the panning procedure, 52.2% (48/92) of clones randomly selected after three panning rounds on InlA were able to bind living *L. monocytogenes* in the screening ELISA using soluble scFv. From these, 30 clones were selected based on signal intensity and sequenced. On the other hand, against InlB, only one binder (1/92) was identified. Interestingly, three of the anti-InlA antibodies originated from the same germline sequences for the variable regions of the heavy and light chains (Table 2). While the only anti-InlB sequence had an Amber stop codon at the beginning of its sequence, which was later corrected for mammalian cell expression.

**Table 2:** Diagnostic performance of four scFv-Fc targeting InlA and one against InlB.

**Target**	**Antibody**	**Germlines**	**Best concentration^a^**	**Sensitivity % (CI)**	**Specificity % (CI)**
InlA	GSM29-D3	VH: IGHV3-30*18 VL: IGLV3-21*02	All three tested	100.0 (29.24–100.0)	100.0 (88.78–100.0)
	GSM29-E6	VH: IGHV3-30*18 VL: IGLV3-21*02	All three tested	100.0 (29.24–100.0)	100.0 (88.78–100.0)
	GSM29-G5	VH: IGHV3-30*18 VL: IGLV3-21*02	All three tested	100.0 (29.24–100.0)	100.0 (88.78–100.0)
	GSM29-H8	VH: IGLV1-51*01 VL: IGHV1-2*02	All three tested	100.0 (29.24–100.0)	100.0 (88.78–100.0)
InlB	GSM30-D2^b^	VH: IGLV2-14*04 VL: IGHV1-3*01	All three tested	100.0 (29.24–100.0)	100.0 (88.78–100.0)

a *The antibody concentrations used were: the EC_50_; a √10-fold concentration above the EC_50_ (EC_50_+); and a √10-fold concentration below (EC_50_-). “Best concentration” refers to the concentration in which the diagnostic performance was superior. In this case, all concentrations showed identical performance*.

b *GSM30-D2 had a stop codon in the VH gene, which was later corrected for the scFv-Fc production*.

*CI, confidence interval*.

This low number of selected scFv against InlB may have some explanations. Since six of the 92 binders were able to bind the recombinant protein, but only one recognized the live cells, this may indicate significant differences in the structure of the antigen when coated (Supplementary Figure S1). Recent studies described considerable differences between panning on plates, used in this study, and in solution, indicating that some proteins might better keep their properties when not exposing their hydrophobic regions for coating (32). The same InlB used in this study showed to be functional in cellular assays from a previous work (25). Another possibility may be due to a low number of human antibody germline sequences against this antigen. Even though it is unlikely that phage display does not provide antibodies against bacterial antigens (33), previous works described regions of bacterial antigens, including *Listeria* spp., that provide few antibodies (23). This indicates that InlB may be substantially different from InlA (identity = 28%), which leads to low number of hits when performing panning. In addition, the fact that the only selected antibody against InlB in this work has a stop codon indicates that some VH germlines against this antigen might have been produced in low amounts or even excluded from the library.

**Figure 1:**
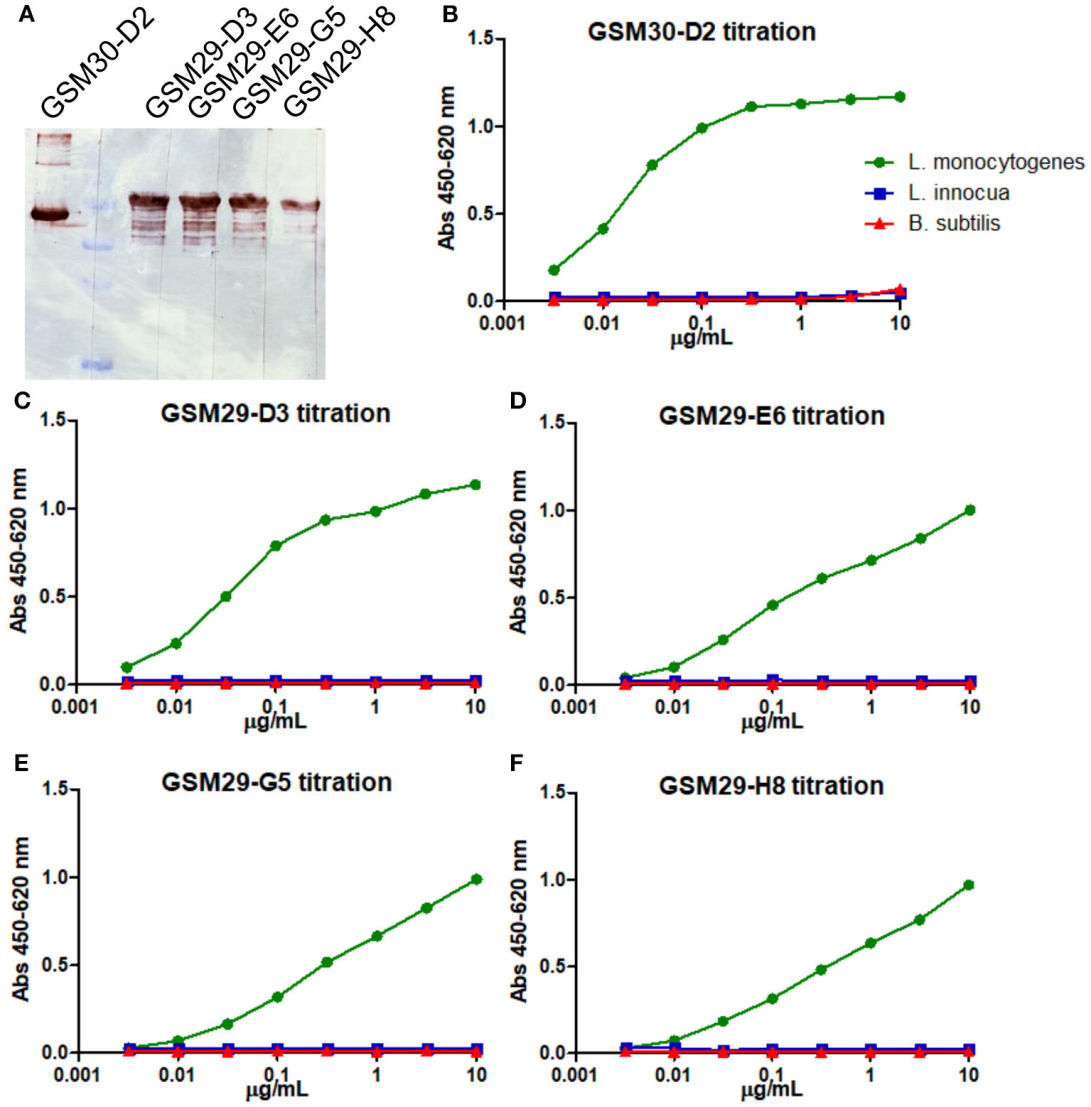
Immunoblot and indirect ELISA for titration and initial specificity screening of the scFv-Fc antibodies against recombinant InlA and InlB. The five mAbs were diluted to 1 μg/mL and tested against 1 μg of the recombinant protein via immunoblot **(A)**. The anti-InlB scFv-Fc GSM30-D2 **(B)**, and the anti-InlA GSM29-D3 **(C)**, GSM29-E6 **(D)**, GSM29-G5 **(E)**, and GSM29-H8 **(F)** were tested against three strains coated alive onto ELISA plates: *L. monocytogenes* ATCC 7644 (green), *L. innocua* DSM 20649 (blue), and *B. subtilis* 168 NCIB 10106 (red).

The four InlA and one InlB binders with unique sequence were produced as scFv-Fc fusions and purified on Protein A-affinity chromatography. These antibodies were initially tested in Western blot against 1 μg of the respective recombinant antigens (Figure 1A). Despite the observation that one of the anti-InlA with different germline sequence showed slightly weaker staining, all antibodies detected bands of the expected molecular mass in immunoblot. This is an indication that the epitopes recognized by these antibodies are resistant to the harsh conditions applied during sample preparation, i.e., boiling and reducing environment. In addition, the scFv-Fc were titrated over *Listeria* living cells (Figures 1B–F), where only one of the four anti-InlA (GSM29-D3) and the anti-InlB (GSM30-D2) reached saturation against living cells. Interestingly, the three mAbs not showing saturation were from the same germline. Although no connection between germline and titration behavior had been established yet, the lack of saturation may help to explain the antibody behavior in different immunoassays. In any case, all five mAbs were specific to *L. monocytogenes* in this initial assay.

### Recombinant scFv-Fc mAbs Are Specific to *L. monocytogens* via Indirect ELISA

**Figure 2:**
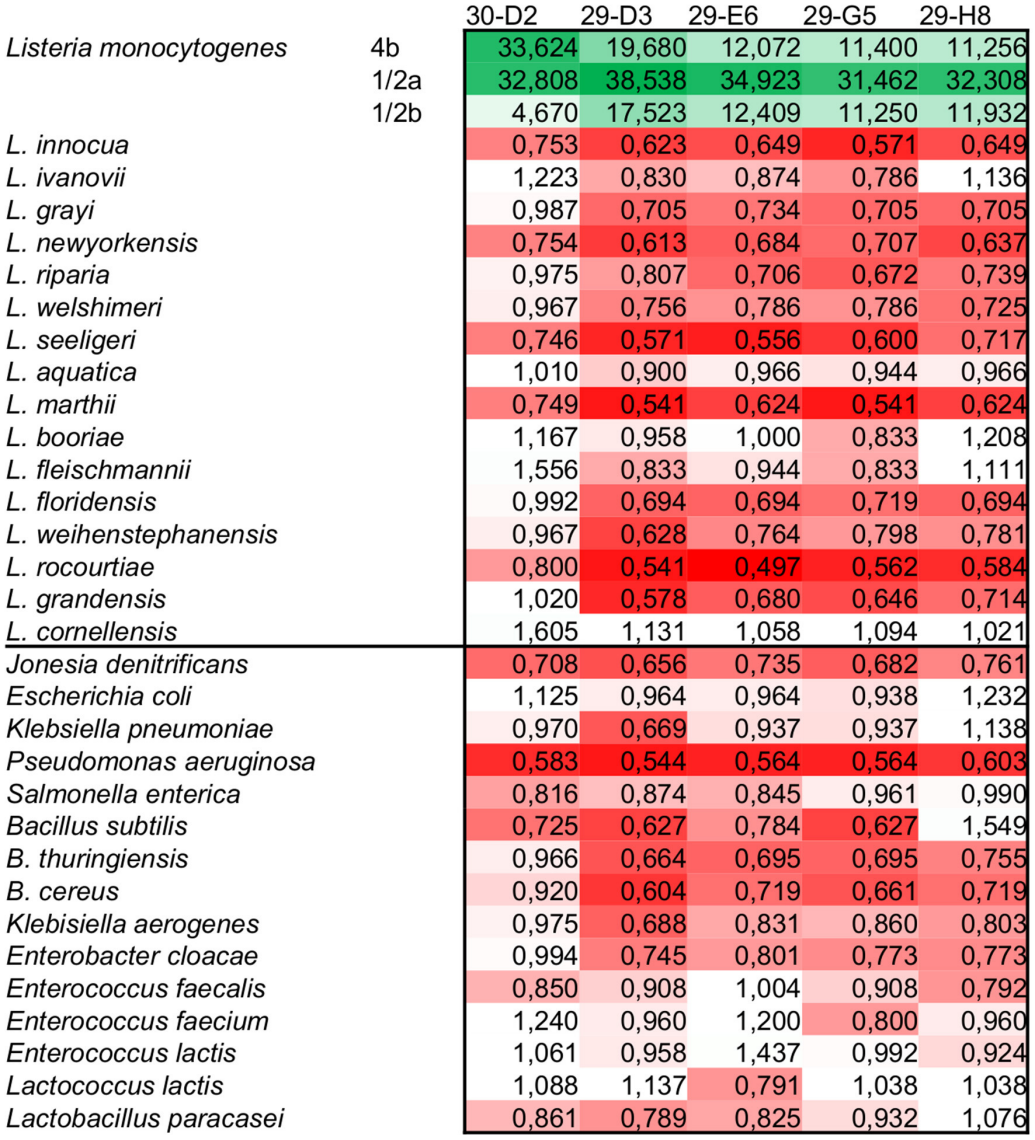
Signal-to-noise ratio of the scFv-Fc tested using the highest concentration (EC_50_+) in indirect ELISA. All five mAbs showed specific reaction against *L. monocytogenes*. Depending on the serotype and target used, the signals were from 4 up to more than 35 times higher than the negative reactions. The color scale goes from green (higher reaction) to red (lower reaction) going through white (signal-to-noise ratio = 1).

The five initially characterized antibodies (four against InlA, and one against InlB) were tested using indirect ELISA against a panel of *Listeria* spp., including the most virulent serovars 4b, 1/2a, and 1/2b. To access the detection performance, a ROC analysis was performed, showing that all five mAbs have 100% sensitivity and specificity (Table 2). When it comes to the reactivity of the antibodies, there is a clear distinction between the detection of InlA and InlB. The four antibodies against InlA showed a signal-to-noise ratio ranges between 11 to 20 for serotypes 4b and 1/2b, and 31 to 38 against 1/2a (Figure 2, Supplementary Table S1). The antibody against InlB had signal-to-noise ratios higher than 30 for serotypes 4b and 1/2a and slightly below 5 against 1/2b. All the non-target strains showed signal-to-noise ratios below 2.

Although the present study could not involve a large panel of *L. monocytogenes* strains, some characteristics in the pattern of antibody reactivity are notable. Both 4b and 1/2b serotypes are part of the genetic lineage I of pathogenic *Listeria* (34), and the reactivity against InlA antibodies proves that both show similar levels of this target on the bacterial surface, while for InlB, serotype 4b shows a much superior amount of this target in comparison to 1/2b serotype. Considering that both serotypes are frequently isolated from human samples, this reactivity correlates to the fact that 4b serotype is highly infective (35, 36). On the other hand, serotype 1/2a is part of genetic lineage II, which is mainly isolated from food samples. However, the strain DSM 102976 strain used in the present study was isolated from an infected Guinea pig. This indicates that contrary to the fact that this serotype is usually less infective than 4b, the high levels of both InlA and InlB showed in the reactivity profile may have been a result of previous infective passages of this strain. Indeed, although these two internalins are the main factors known to promote intestinal cells invasion, other genes involved in the pathogenesis and survival of *Listeria* are important to determine virulence (37–39). Other environmental factors, such as temperature, may also influence the expression level of these targets, although the culture conditions used in the present study were the most appropriate for growth and both InlA and InlB in general (40).

Both InlA and InlB are the most studied proteins for *L. monocytogenes* detection. In other studies, mAbs against InlA were generated by hybridoma technology and were able to provide molecules with variable sensitivity. When using different highly sensitive biosensors, some works described the limit of detection (LOD) of 10^7^ cells/mL (41), while others described 3 x 10^2^ CFU/mL (24). An aptamer binder against InlA allowed detecting 10^3^ CFU/mL when combined with other mAbs in SPR (42). Regarding InlB, a previous work described scFv binders that could be used for *L. monocytogenes* staining with quantum dots, as well as anti-InlA scFv (43). Phage display has also been previously used to obtain VHH antibodies from llamas immunized with InlB, although the applicability of this nanobody in immunodetection was not further investigated (44). Binders toward other targets were described, such as the hybridoma-derived mAbs against LapB, which were able to recognize more than 46 of the 53 *L. monocytogenes* strains included in the experiment (45). The use of a phage display human synthetic antibody library to generate antibodies against *L. monocytogenes* resulted in one scFv targeting ActA found to specifically recognize 6 out of 8 tested pathogenic strains (46). This same molecule was also tested in SPR showing LOD of 2 x 10^6^ CFU/mL (47). Nonetheless, similarly to the VHH generated via phage display, this molecule was not further assessed for the development of a detection immunoassay. Considering that commercial lateral flow tests are already able to detect 10^4^-10^6^ CFU/mL, all the newly generated antibodies offer a chance for improving the minimal amount of *Listeria* cells detected.

In the present study, the generation of recombinant antibodies from a human naïve antibody library via phage display against InlA and InlB is described. Taking this into account, although the main goal was to generate a detection method for *L. monocytogenes*, the fully human origin of these antibodies would also facilitate the therapeutic application of these mAbs against *Listeria* infection in humans. This is especially important considering that, even though listeriosis has a low incidence overall, its mortality rate can be as high as 25%, with most outbreaks describing more than 10% mortality (48). Moreover, the increasing number of *Listeria* isolates that are resistant to antibiotics is already causing concern in the medical community (49). In this aspect, antibodies may offer an interesting alternative to antibiotics in the near future. Indeed, mAbs against Ferritin-like protein (Flp), ActA, or Listeriolysin O (LLO) have shown promising results in protecting mice against *Listeria* infection (50, 51). Thus, the recombinant human mAbs presented here could be an interesting alternative for passive immunization to reduce the number of deaths caused by listeriosis.

The panel of *L. monocytogenes* strains used here included the three most prevalent serovars in clinical cases (34). Besides that, no cross-reaction against any of the other 31 tested species was observed, indicating the high applicability of these antibodies for detection. As to the characteristics of *L. monocytogenes*, it is known that some isolates contain either truncated or mutated InlA or InlB (52, 53). Even though the number of isolates included in the study was low, no relevant strain containing both antigens altered has ever been described, indicating that the combination of mAbs targeting InlA and InlB still allow the detection of such strains. Therefore, the recognition profile of the generated mAbs described here indicates they have high differentiation capability, allowing the specific detection of *L. monocytogenes* in further studies with a detection procedure which is close to the final detection method (e.g., lateral flow). In this way, the current study indicates that the use of the anti-InlA e InlB mAbs in combination with those previously described against PDC-E2 could allow the proper detection of both pathogenic and non-pathogenic *Listeria* species.

## Conclusion

Considering the growing importance of the genus *Listeria* in recent years, it is essential to increase studies aiming to develop tools to identify and control it. Since listeriosis is a foodborne disease that can be highly fatal, proper testing of food and food production facilities is the best way to prevent outbreaks. Hence, the present work shows the use of antibody phage display to generate suitable antibodies that can further improve the detection of *L. monocytogenes*. In future works, it might be possible to combine them with broadly-specific antibodies allowing the detection of the entire *Listeria* genus to create an immunoassay that differentiates between pathogenic and non-pathogenic *Listeria*.

## Data Availability Statement

The datasets presented in this study can be found in online repositories. The names of the repository/repositories and accession number(s) can be found at: https://publikationsserver.tu-braunschweig.de/receive/dbbs_mods_00066984.

## Author Contributions

GM, MM, ÂM, FC, and MH: conceptualization and funding acquisition. GM: investigation and writing—original draft. GM, SG, SD, MM, FC, and MH: methodology and writing—editing. SG, FC, ÂM, SD, and MH: resources. SD, MM, FC, and MH: supervision. All authors contributed to the article and approved the submitted version.

## Conflict of Interest

SG was employed by German Collection of Microorganisms and Cell Cultures GmbH (DSMZ). The remaining authors declare that the research was conducted in the absence of any commercial or financial relationships that could be construed as a potential conflict of interest.

## Publisher's Note

All claims expressed in this article are solely those of the authors and do not necessarily represent those of their affiliated organizations, or those of the publisher, the editors and the reviewers. Any product that may be evaluated in this article, or claim that may be made by its manufacturer, is not guaranteed or endorsed by the publisher.
