# Supplementary material for: Phage Display-Derived Monoclonal Antibodies Against Internalins A and B Allow Specific Detection of Listeria monocytogenes
Source: Front Public Health. 2022 Mar 15;10:712657. doi: 10.3389/fpubh.2022.712657 (PMC8964528; doi:10.3389/fpubh.2022.712657)
Supplement: Supplementary file 2 [file Table_2.DOCX]

Supplementary Material


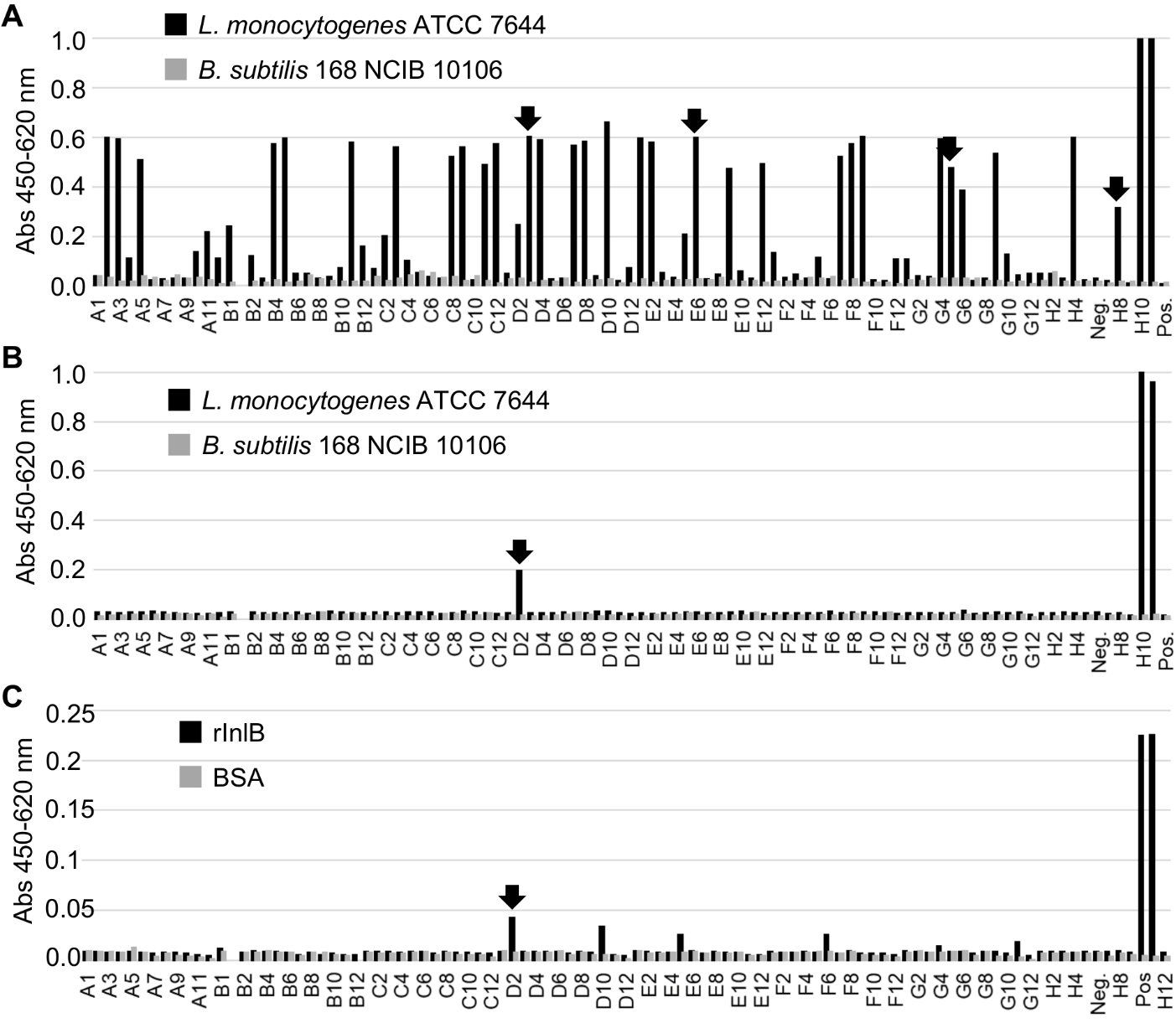


**Supplementary Figure 1.** Screening results after performing the panning over recombinant InlA or InlB. From each of the four panning strategies, 92 clones were tested against the respective protein in parallel with BSA, and different bacteria (*L. monocytogenes* ATCC 7644, and *B. subtilis* 168 NCIB 10106). The figure shows the positive (black bars) and negative (gray bars) antigens. Even though some of the presented graphics show more reactive antibodies than the selected (black arrows), they were discarded mainly because they had the same sequence. (A) Screening results from panning using InlA, which led to four binders. (B) Screening results from panning using InlB, which led to one binder against *L. monocytogenes* cells. (C) Screening results from panning using InlB, which led to six binders, but only the one recognizing cells is marked with the black arrow.

**Supplementary File 1.** Reads from the ELISA plates used for the determination of detection performance. Each sheet corresponds to one different bacterial species. Only the reads of the wells used in the present study are shown.
